# Failure Load and Fatigue Behavior of Monolithic Translucent Zirconia, PICN and Rapid-Layer Posterior Single Crowns on Zirconia Implants

**DOI:** 10.3390/ma14081990

**Published:** 2021-04-15

**Authors:** Frank A. Spitznagel, Sara Röhrig, Robert Langner, Petra C. Gierthmuehlen

**Affiliations:** 1 Department of Prosthodontics, Medical Faculty and University Hospital Düsseldorf, Heinrich-Heine-University, 40225 Düsseldorf, Germany; sara.roehrig@med.uni-duesseldorf.de (S.R.); petra.gierthmuehlen@med.uni-duesseldorf.de (P.C.G.); 2 Institute of Systems Neuroscience, Medical Faculty, Heinrich-Heine-University, 40225 Düsseldorf, Germany; robert.langner@uni-duesseldorf.de; 3 Institute of Neuroscience and Medicine, Brain and Behaviour (INM-7), Research Centre Jülich, 52425 Jülich, Germany

**Keywords:** dental implant, zirconia, ceramics, translucent zirconia, fatigue, failure load, aging, chewing simulation

## Abstract

This laboratory study aimed to evaluate the thermo-mechanical fatigue behavior and failure modes of monolithic and rapid-layer posterior single-crowns (SCs) supported by zirconia implants. Methods: 120 all-ceramic crowns supported by one-piece zirconia implants (ceramic.implant; vitaclinical) were divided into five groups (*n* = 24 each): Group Z-HT: 3Y-TZP monolithic-zirconia (Vita-YZ-HT); Group Z-ST: 4Y-TZP monolithic-zirconia (Vita-YZ-ST); Z-XT: 5Y-TZP monolithic-zirconia (Vita-YZ-XT); Group E: monolithic-polymer-infiltrated ceramic network (PICN,Vita-Enamic); Group RL (rapid layer): PICN-“table-top” (Vita-Enamic), 3Y-TZP-framework (Vita-YZ-HT). Half of the specimens of each group (*n* = 12) were exposed to fatigue with cyclic mechanical loading (F = 198N, 1.2-million cycles) and simultaneous thermocycling (5–55 °C). Single-load-to-failure testing (Z010, Zwick) was performed for all specimens without/with fatigue application. Data analysis was performed using ANOVA, Tukey’s post-hoc test, two-sample t-test and Bonferroni correction (*p* < 0.05). Results: All specimens survived fatigue exposure. Significant differences in failure loads were detected among groups (*p* ≤ 0.004). Materials Z-HT and Z-ST showed the highest failure loads followed by Z-XT, RL and E. The influence of fatigue was only significant for material RL. Conclusions: All types of tested materials exceeded clinically acceptable failure load values higher than 900N and can be recommended for clinical use. Z-HT and Z-ST appear to be highly reliable towards fatigue. Rapid-layer design of PICN and YZ-HT might be an interesting treatment concept for posterior implant SCs.

## 1. Introduction

Today, ceramic implants can be considered a valid treatment addendum in the portfolio of dental implantology with osseointegration capacities and survival rates similar to titanium implants [1,2,3]. Encouraging survival rates of 98.4–100% with marginal bone losses of 0.7–1.2 mm are reported after 5–7.8 years of loading for zirconia implants [3,4]. Recent pre-clinical and clinical trials suggest favorable soft tissue responses regarding biofilm formation, plaque scores and experimental mucositis conditions of zirconia implants [5,6,7]. Patient wishes towards metal-free solutions and a growing market for ceramic implants is expected to further increase the demand for zirconia implants in the near future [8,9]. 

As most of the studies solely focus on soft- and hard-tissue integration of zirconia implants, little evidence is published on the long-term behavior of their prosthetic counterparts [10,11]. However, both the implant and the restorative crown-abutment complex have to be considered as one unit when evaluating a successful dental implant treatment. Recent clinical studies that evaluated the mid-term behavior of single crowns and fixed-dental prostheses on ceramic implants reported high incidences of technical complications, especially chipping events for bi-layer systems of up to 47% after five years [11,12,13]. 

To overcome the risk of chipping for veneered restorations, an increased application of monolithic materials can be observed in fixed tooth- and implant prosthodontics [14,15]. Reduced fabrication expenses and time, high reliability and survival rates with less technical complication rates are considered to be the main advantages and improvements of monolithic restorations [16,17]. 

Besides reinforced glass ceramics, resin-matrix ceramics (RMC) and zirconia ceramics are considered to be suitable materials for monolithic application. So far, zirconia was mainly used as a framework material due to its high flexural strength but opaque appearance [17]. Recently, developments and improvements of mechanical and optical properties have led to more translucent zirconia ceramics [17,18]. An increase in translucency could be achieved by reducing the concentration of alumina additives and using higher yttria contents (4Y-, 5Y-TZP) with increased contents of cubic phase [18,19]. However, the enhancement of optical properties led to a reduction in strength and toughness [18,20].

CAD/CAM RMCs and especially polymer-infiltrated ceramic networks (PICN) were introduced as more flexible and elastic materials, combining positive material properties of ceramics and polymers [21,22]. Due to its dual network and a dentin-like modulus of elasticity, PICN could potentially absorb and distribute occurring occlusal forces more favorably, making it an interesting restorative material for implant prosthodontics [23,24]. 

Yet, neither in vitro nor clinical studies analyzing the performance of monolithic translucent zirconia restorations or a restorative combination of PICN with zirconia on ceramic implants could be identified. Hence, the performance of these materials on ceramic implants remains to be elucidated. 

Therefore, the purpose of this in vitro study was to evaluate the thermo-mechanical fatigue behavior and failure modes of monolithic and rapid-layer posterior translucent zirconia and PICN single crowns supported by one-piece zirconia implants. The tested null hypotheses assumed: (i) type of restoration material and (ii) fatigue exposure do not significantly influence the failure load of posterior all-ceramic implant crowns. Monolithic 3Y-TZP implant restorations served as control. 

## 2. Materials and Methods

**Figure 1:**
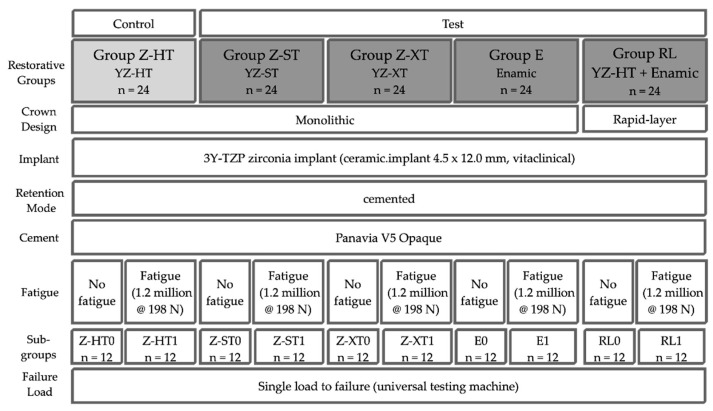
Study set-up.

A total of 120 one-piece zirconia implants (3Y-TZP, ceramic.implant 4.5 mm × 12.0 mm, vitaclinical, Bad Säckingen, Germany) were divided into four test groups and one control group (*n* = 24 specimens each) according to the type of restoration material (Figure 1 and Table 1):

- Control group Z-HT: 3Y-TZP monolithic “high translucent” zirconia crown (Vita YZ-HT, Vita Zahnfabrik, Bad Säckingen, Germany);
- Test group Z-ST: 4Y-TZP monolithic “super translucent” zirconia crown (Vita YZ-ST, Vita Zahnfabrik);
- Test group Z-XT: 5Y-TZP monolithic “extra translucent” zirconia crown (Vita YZ-XT, Vita Zahnfabrik);
- Test group E: monolithic polymer-infiltrated ceramic network crown (PICN, Vita Enamic, Vita Zahnfabrik);
- Test group RL (rapid layer): polymer-infiltrated ceramic network (PICN Vita Enamic, Vita Zahnfabrik) “table top” adhesively bonded to a 3Y-TZP framework (Vita YZ-HT, Vita Zahnfabrik).

### 2.1. Fabrication of Implant Crowns

**Figure 2:**
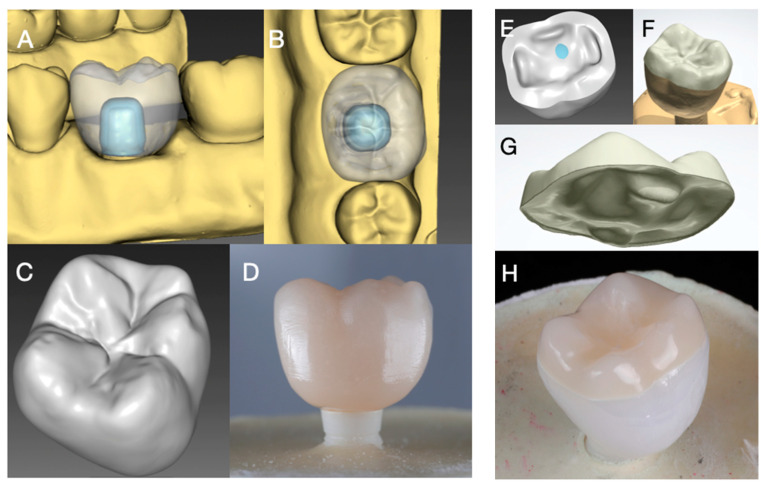
Digital restoration design: (**A**–**C**) master design for monolithic groups and (**D**) monolithic specimen; (**E**–**G**) split design of crown into 3Y-TZP (**E**) framework and PICN table top (**G**) of Group RL (**F**,**H**).

For standardization, the zirconia implant was embedded in the position of a mandibular first molar in a prosthetically optimal position in a master model (Frasaco Model, Frasaco, Tettnang, Germany). The master model was scanned (InEos X5, Dentsply Sirona, York, PA, USA) and a master design of a mandibular molar crown (InLab 18.1, Dentsply Sirona) was used for all monolithic crowns to ensure identical and comparable test specimens (Figure 2A–D). The master crown design for Group RL was split to generate a separate framework and veneer layer (Figure 2E–H). All implant restorations were produced in a commercial dental laboratory by the same master dental technician according to manufacturer’s recommendation. All restorations were milled in a five-axis milling machine (inLab MC X5, Dentsply Sirona). 

### 2.2. Preparation of Specimens

All zirconia implants (Group Z-HT, Z-ST, Z-XT, E and RL) were perpendicularly embedded in polyester resin (Technovit 4000, Kulzer, Hanau, Germany) with a simulation of 0.5–1 mm bone resorption between the implant neck and resin surface. Hence, clinical conditions with reported marginal bone losses of 0.70–0.79 mm after 1 year [2,25] and 0.7 ± 0.6 mm 5 years after loading were represented [3].

### 2.3. Adhesive Cementation

PICN implant restoration of Group E and PICN “table top” of Group RL were first cleaned with 70% ethanol and pretreated with 5% hydrofluoric acid (Vita Ceramics Etch, Vita Zahnfabrik, Bad Säckingen, Germany), rinsed with air–water spray (30 s) and afterwards subjected to ultrasonic bath (distilled water; 5 min) and air-dried. 

The intaglio crown surface of Group Z-HT, Z-ST and Z-XT were first air-particle abraded with 50 μm aluminum oxide at a pressure of 2 bar prior to cementation. The zirconia framework of Group RL was treated accordingly. All zirconia restorations (Z-HT, Z-ST, Z-XT and the zirconia framework of Group RL) were afterwards ultrasonically cleaned with 70% ethanol for 3 min and chemically modified with an MDP-containing primer (Clearfil Ceramic Primer Plus, Kuraray Noritake, Tokyo, Japan). PICN table tops of Group RL were adhesively luted with a self-curing resin composite cement (Panavia V5 opaque, Kuraray Noritake) to the pretreated zirconia substructure. The primer (Clearfil Ceramic Primer Plus) was also applied to PICN restorations and to the implant abutment of the investigated one-piece ceramic implant. Table 2 summarizes the pretreatments and adhesive cementation of all tested materials. All implant restorations were afterwards bonded with a resin composite cement (Panavia V5 opaque) to the zirconia implants in the self-curing mode. 

### 2.4. Fatigue Analysis

Directly after cementation, half of the specimens of each group (*n* = 12 each) were exposed to cyclic mechanical loading and simultaneous thermocycling (5 °C to 55 °C, dwell time 120 s) in a chewing simulator (CS-4.8 professional line, SD Mechatronik, Feldkirchen-Westerham, Germany). An occlusal load of 198 *n* at 1.2 million chewing cycles with a frequency of 1.6 Hz was applied to the mesio-lingual cusp of the restoration, simulating a clinical 5-year exposure under clinically relevant conditions [26,27,28]. Force transmission was performed by sliding a steatite indenter (r = 3 mm, Hoechst CeramTec, Wunsiedel, Germany) 0.5 mm down the mesio-lingual cusp towards the central fossa, with a vertical movement of 2 mm for each chewing cycle. During cyclic loading specimens were inspected twice a day for crack and/or fracture failures as well as for mobility of the prosthetic suprastructure. To accomplish 1.2 million chewing cycles approximately 9 days were needed.

### 2.5. Load to Failure

All specimens (fatigued and non-fatigued) were subjected to a single-load-to-failure (SLF) assessment in a universal testing machine (Zwick Z010/TN2S, Zwick Roell, Ulm, Germany). A steel ball with a diameter of 6 mm served as an indenter and was aligned at the same contact point as the dynamic loading with a crosshead speed of 1.5 mm/min. Fractures of the ceramic restoration (cohesive chipping fractures and/or catastrophic bulk fractures) and extensive cracks within the restoration were defined as failure.

### 2.6. Fractographic Analysis

Test samples were visually examined under a digital microscope at a 20-fold magnification (VHX-950F, Keyence, Osaka, Japan) after fatigue and SLF to determine the mode of failure. Representative photographs of fractured specimens were taken. 

Furthermore, most representative specimens of each group were inspected for qualitative fractographic analysis with scanning electron microscopy (SEM, EVO10, Carl Zeiss, Oberkochen, Germany).

### 2.7. Statistical Analysis

Data were statistically analyzed with SPSS 25 (IBM Corp., Armonk, NY, USA). The Levene test was applied to test homogeneity of variance before using ANOVA to test for main effects and interactions of the two factors of interest (type of restorative material and fatigue), followed by Tukey’s post-hoc tests for the pairwise comparison of restorative material types. Influence of fatigue was separately tested for each type of restorative material via two-sample t-tests. The level of significance was set at *p* < 0.05 for all tests, Bonferroni corrected for multiple comparisons where applicable. Data were graphically presented in boxplots.

## 3. Results

### 3.1. Fatigue Exposure

**Figure 3:**
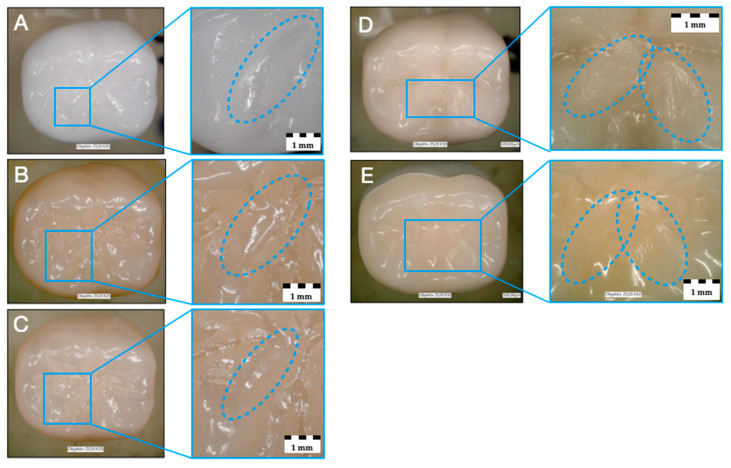
Monolithic zirconia materials after fatigue with superficial and neglectable wear: (**A**) 3Y-TZP (Z-HT), (**B**) 4Y-TZP (Z-ST), (**C**) 5Y-TZP (Z-XT); Materials E (**D**) and RL (**E**) with wear facets after fatigue.

All tested specimens withstood thermo-mechanical fatigue application, resulting in a simulated 5-year survival rate of 100%. No bulk or cohesive fractures within the crown restorations or implants occurred during and after mouth-motion fatigue. No extensive crack formation within the crown restorations or implants was notified. Only superficial wear was observed for the investigated materials Z-HT, Z-ST and Z-XT. Materials E and RL revealed clear wear facets of the PICN restoration material on the loading area of the mesio-lingual cusp (Figure 3).

### 3.2. Single Load to Failure

**Figure 4:**
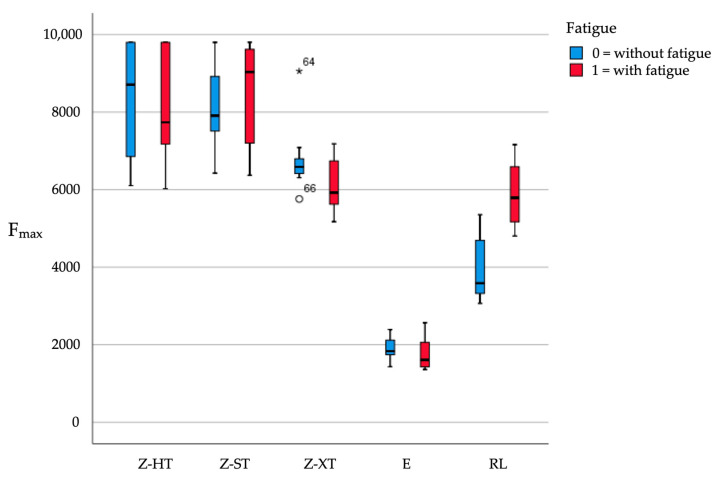
Boxplots of the failure load values (in N) of the tested groups (* indicates outlier for Z-XT).

Failure loads after static loading are presented in Table 3 and illustrated in Figure 4.

Fatigue did not have a significant main effect across restorative material types [F(1,120) = 2.05, *p* = 0.156], but the main effect of restoration material [F(4,120) = 185.93, *p* < 0.001] and its interaction with fatigue [F(4,120) = 6.47, *p* < 0.001] were highly significant.

To further elucidate the interaction, t-tests for the pairwise comparison between specimens with and without fatigue exposure for each restorative material type were performed. The only significant (Bonferroni corrected) difference was found for RL, which showed significantly higher (*p* < 0.001) failure loads with fatigue (Table 3). No significant fatigue effects were found for restorative materials Z-HT (*p* = 0.607), Z-ST (*p* = 0.414), Z-XT (*p* = 0.047) and E (*p* = 0.413). 

The highest mean failure load was detected for group Z-HT0 (8450 N) without fatigue (Z-HT0>Z-ST0>Z-XT0>RL0>E0) and for group Z-ST1 (8557 N) with fatigue (Z-ST1>Z-HT1>Z-XT1>RL1>E1). Material E showed the lowest mean failure load of all tested restorative materials both without and with fatigue application.

Tukey’s post-hoc tests revealed that restorative materials Z-HT and Z-ST showed significantly higher failure loads relative to all other three restorative materials tested (all *p* ≤ 0.004), irrespective of fatigue, but there was no significant difference between Z-HT and Z-ST (*p* = 1).

Restorative materials Z-XT, E and RL also showed highly significant differences in failure load between each other, both without and with fatigue (all *p* < 0.001) (Table 3). The only exception to this remained the subgroup analysis of Z-XT1 versus RL1 (*p* = 1) where no significance was found for fatigue (Table 3).

### 3.3. Failure and Fractographic Analysis after Single-Load-To-Failure Testing

**Figure 5:**
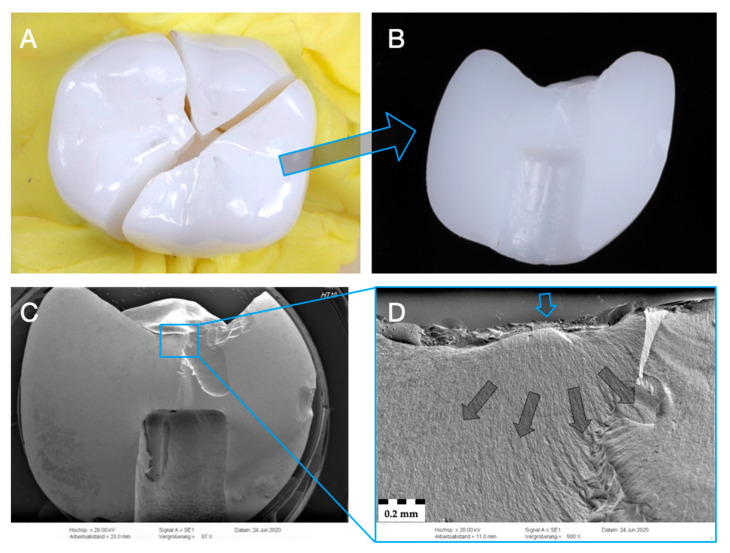
Representative specimen of Group Z-HT after single-load-to-fracture assessment. Light microscopy shows (**A**) occlusal view of bulk fractured specimen and (**B**) side view of fractured part marked with blue arrow. SEM pictures: (**C**) overview of fractured part at low magnification of (**B**). Detailed view at high magnification (**D**): blue arrow indicates fracture initiation point, while dark arrows represent the direction of crack propagation.

**Figure 6:**
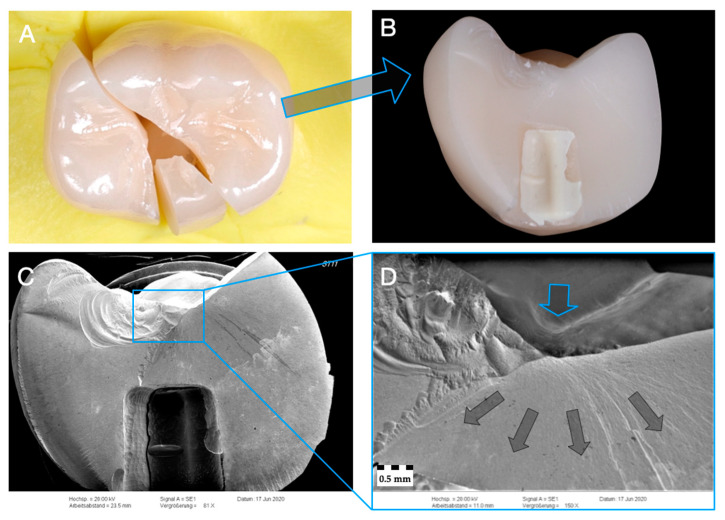
Representative specimen of Group Z-ST after single-load-to-fracture assessment. Light microscopy shows (**A**) occlusal view of bulk fractured specimen and (**B**) side view of fractured part marked with blue arrow. SEM pictures: (**C**) overview of fractured part at low magnification of (**B**). Detailed view at high magnification (**D**): blue arrow indicates fracture initiation point, while dark arrows represent the direction of crack propagation.

**Figure 7:**
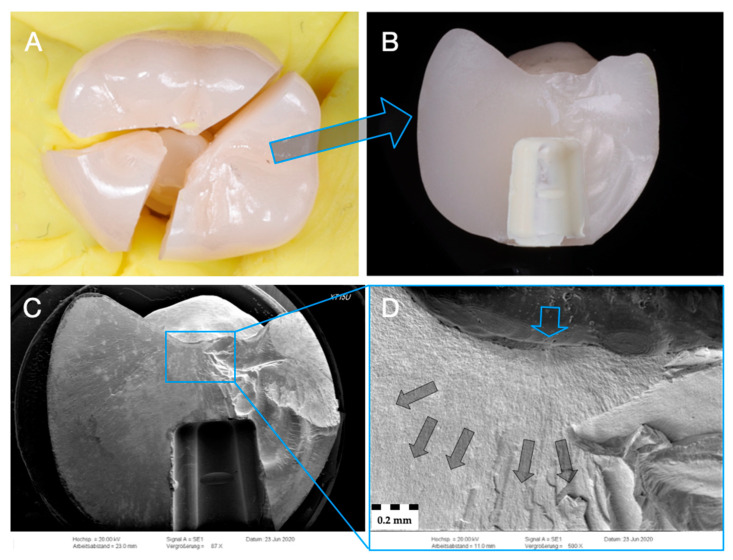
Representative specimen of Group Z-XT after single-load-to-fracture assessment. Light microscopy shows (**A**) occlusal view of bulk fractured specimen and (**B**) side view of fractured part marked with blue arrow. SEM pictures: (**C**) overview of fractured part at low magnification of (**B**). Detailed view at high magnification (**D**): blue arrow indicates fracture initiation point, while dark arrows represent the direction of crack propagation.

**Figure 8:**
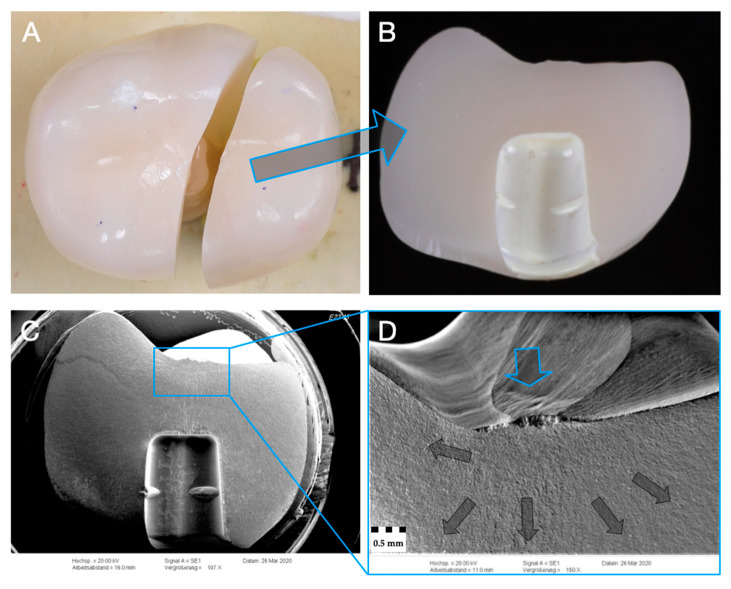
Representative specimen of Group E after single-load-to-fracture assessment. Light microscopy shows (**A**) occlusal view of bulk fractured specimen and (**B**) side view of fractured part marked with blue arrow. SEM: (**C**) overview of fractured part at low magnification of (**B**). Detailed view at high magnification (**D**): blue arrow indicates fracture initiation point, while dark arrows represent the direction of crack propagation.

**Figure 9:**
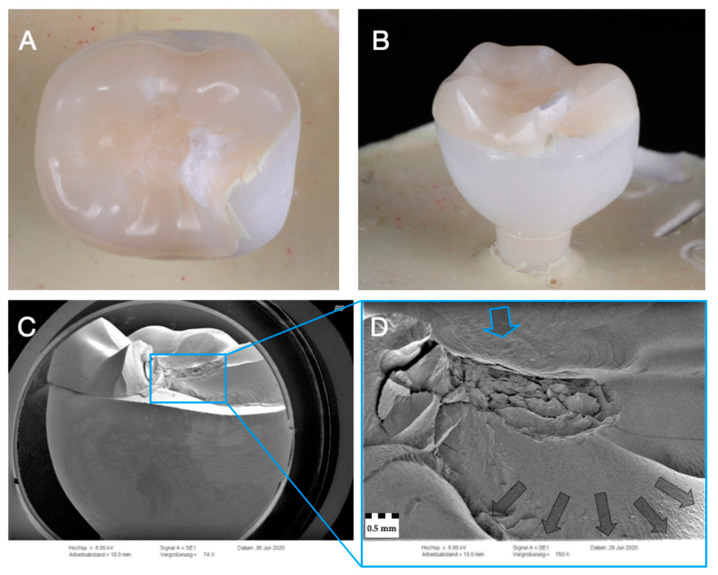
Representative specimen of Group RL after single-load-to-fracture assessment. Light microscopy shows (**A**) occlusal view of PICN table top chipping and (**B**) side view of fractured part. Note the intact underlying zirconia framework. SEM pictures: (**C**) overview of chipped PICN table top at low magnification with intact zirconia substructure. Detailed view at high magnification with complex crack course (**D**): blue arrow indicates fracture initiation point, while dark arrows represent the direction of crack propagation.

All monolithic crowns of Z-XT and E revealed catastrophic bulk fractures after single load to failure. Monolithic zirconia crowns of Z-HT and Z-ST predominately suffered from catastrophic bulk fractures. Cracks occurred on four specimens of material Z-HT and on one of Z-ST. Both subgroups of material RL (RL0 and RL1) solely showed fractures of the PICN table top, whereas the underlying zirconia framework revealed no fractures (Figure 5A,B, Figure 6A,B, Figure 7A,B, Figure 8A,B and Figure 9A,B).

For all monolithic materials (Figure 5C,D, Figure 6C,D, Figure 7C,D and Figure 8C,D), fractographic SEM analysis revealed telltale markings such as hackles depicting the fracture origin initiating at the occlusal load area. Hackles are lines on the ceramic surface indicating the direction of crack propagation. 

In contrast, for material RL (Figure 9C,D), fractographic analysis showed a complex crack course in different directions but mainly towards the adhesive interface between the PICN table top and the zirconia substructure. Of note, there was no fractures of the underlying zirconia framework. 

## 4. Discussion

This in vitro study investigated the failure load and fatigue behavior of different designs of monolithic translucent zirconia, PICN and rapid-layer posterior implant crowns on one-piece zirconia implants. The tested null hypothesis was rejected as (i) type of restoration material and (ii) fatigue application in part (only for RL) affected the failure load.

Dynamic loading and accelerated aging tests are a reliable method to provide essential information on limitations and lifetime predictions of ceramic restorations [29]. The applied fatigue protocol with 1.2 million cycles and simultaneous thermocycling corresponds to a clinical exposure time of 5 years and is a well-accepted method [27,28,30,31,32]. In the present study, all specimens survived exposure to an artificial oral environment with thermomechanical loading. All implant crowns, with and without fatigue application, showed mean failure loads above 1750 N after single load to failure and exceeded reported physiological occlusal forces of 200–900 N in the posterior dentition [33]. These findings suggest that all tested monolithic and rapid-layer implant crown designs are suitable for clinical application on one-piece zirconia implants in the molar area from a mechanical point of view. 

The highest failure loads were recorded for monolithic 3Y-TZP (Z-HT) and 4Y-TZP (Z-ST) zirconia crowns due to their high flexural strength and fracture toughness [19]. The mean failure loads of both groups were almost nine times higher (>8140 N) than normal chewing forces. No statistically significant difference (*p* = 1) of failure loads between 3Y-TZP and 4Y-TZP monolithic zirconia ceramics was found. Monolithic 4Y-TZP zirconia (Z-ST) could therefore be a preferable choice of material for monolithic posterior implant-supported single crowns, since it seems as durable as 3Y-TZP but with better translucency and esthetics [34]. 

In vitro studies reported loading capacities for 3Y-TZP monolithic zirconia crowns (Vita YZ T) above 6000 N [30] on one-piece zirconia implants from the same manufacturer (vitaclinical) and between 4700 and 6350 N for 3Y-TZP monolithic implant crowns (Lava Plus) on one-piece zirconia implants (PURE Ceramic Implant, Straumann) [31] and 6310 N for 3Y-TZP monolithic zirconia crowns (Zerion LT) on two-piece zirconia implants (PURE two-piece implant, Straumann) [32]. Identical fatigue protocols simulating 5 years of clinical service was applied in the aforementioned studies to further stress the restorative-implant complex [30,31,32]. Different ranges in failure loads can be explained by different implant diameters (4.1 mm), two-piece design [32], abutment height (4.0 mm) [31] and choice of adhesive cements [30], as well as the crown design itself. Furthermore, it must be noted that the small geometry of the one-piece implant abutment results in an increased wall thickness of the restoration and, consequently, leads to higher failure loads compared to restorations based on individually designed or prefabricated abutments [30]. 

Comparable failure loads of 1162 N [35], 1297 N [36] (both implant diameter of 4.0 mm) and up to 1591 N [30] (implant diameter 4.5 mm) were reported for aged and non-aged monolithic PICN implant crowns on one-piece ceramic implants from the same manufacturer (vitaclinical). Fatigue did also not influence the failure load of PICN in the aforementioned study [30]. PICN and feldspar implant crowns bonded to one-piece ceramic implants benefit from MDP-containing adhesive cements [30,37], while no direct correlation could be found for zirconia crowns [31]. However, in general, resin bonding is also considered beneficial for zirconia restorations and can extend their lifetime estimation [38,39]. Recently published in vitro studies confirm the beneficial effect of adhesive cementation and proper surface treatment to translucent zirconia [40,41]. 

A significant influence of fatigue was observed only for PICN/3Y-TZP rapid-layer implant crowns, where fatigue significantly increased after thermomechanical loading. 

A possible reason of higher failure loads for fatigued rapid-layer PICN/3Y-TZP implant crowns of group RL1 compared to RL0 might be a complete polymerization of the adhesive resin interface during thermomechanical loading resulting in a strength increase and durable bond between the PICN and the underlying 3Y-TZP (YZ-HT) framework. In general, a 1-day water storage at 37 °C prior to thermal cycling is recommended to allow a complete polymerization of the adhesive interface [42]. 

Failure and fractographic analysis revealed that under load, all monolithic crowns were stretched apart with a fracture onset probably starting in the fissure. In the fissure, the material is subjected to tensile stresses, which is the most unfavorable loading case and leads finally to a bulk fracture. 

In contrast, material RL shows a different loading situation and fracture pattern. The PICN table tops are predominately loaded under pressure and the adhesive interface is more or less loaded in a right angle, resulting in slight shearing between the base of the PICN table top and the underlying 3Y-TZP framework. This loading scenario already leads to a high initial failure load, since the PICN table top is well supported by the 3Y-TZP zirconia framework and is primarily loaded under pressure.

If now the polymerization of the resin cement continues during the chewing simulation, this situation is improved by two effects: (1) the compressive strength of the luting material increases and better supports the PICN table tops [36]; (2) shear at the interface becomes more difficult as the luting material strengthens. This assumption is assisted by an in vitro study where the compressive strength of the resin cement also increased significantly after autopolymerization and thermocycling within the first few days [42]. The observed fracture pattern with chipping but without detachment of the PICN table tops further supports this assumption. However, further studies might investigate the strength increase in fatigued and non-fatigued RL restorations with regard to water storage prior to thermal cycling and continued polymerization of the resin cement. 

Time-dependent fatigue fractures can occur due to repetitive cyclic loading, which leads to subcritical crack propagation and ultimately to restoration failure [43]. Occlusal wear scars and facets are often considered the trigger point for fatigue fractures [43,44]. After fatigue, wear facets were noted for PICN, whereas for monolithic zirconia only superficial and neglectable wear could be observed. PICN shows a similar wear behavior to enamel, but significant reduction in surface gloss and more wear than feldspathic ceramics are reported [45]. Likewise, observations with substantial wear for PICN crowns on zirconia implants after thermomechanical loading were made [24]. The authors attributed the increased wear behavior of PICN to its elastic properties and the rigid zirconium implant. As a result, occurring occlusal forces could only be absorbed by the restorative material itself and ultimately lead to increased wear of the latter [24]. However, no fractures or catastrophic failures were observed after fatigue similar to the present study [24]. 

Translucent 4Y- and 5Y-TZP ceramics showed a similar wear behavior to 3Y-TZP zirconia in a recent in vitro study [46], which is in line with the observations of this study. However, zirconia surfaces have to be properly finished to be wear resistant and antagonist friendly. Polished zirconia showed the least wear and best antagonist behavior both in vitro [47,48] and in clinical studies [49]. 

One limitation of the current study is the laboratory design itself, which cannot fully represent the clinical environment. Moreover, load-to-fracture tests have been criticized in the past [50,51], and wear behavior was not evaluated qualitatively. 

So far, no comparable data for the proposed rapid-layer design on ceramic implants are available. Comparing materials E and RL, it seems that the underlying zirconia framework reinforced and doubled the failure loads. This restorative combination could merge the advantages of the PICN material with a high-strength 3Y-TZP or 4Y-TZP substructure. The PICN could offer the benefits of a tooth-like wear behavior [52], an ability to absorb occurring occlusal forces, which prevents overloading of the implant–bone complex and protects the implant [24], and, in the case of fatigue failures, an easy and fast possibility of replacement as it is designed as an occlusal table top. The zirconia substructure could serve as an esthetic high-strength support with preferred biological responses to the surrounding peri-implant soft tissue [53,54,55]. This proposed combination of a high-strength zirconia framework and a PICN material on top might be an interesting and novel restorative treatment concept for both zirconia and titanium implants in the posterior dentition, warranting further research especially when monolithic implant-supported zirconia restorations serve as antagonists. As the design of the PICN table top is digitally achieved in the CAD/CAM system, it can be easily replaced in case of increased wear or fracture. 

## 5. Conclusions

Based on the present results and within the limits of this in vitro study, the following conclusions can be drawn:

1. Fatigue did only influence the failure load of the restorative concept RL (higher failure loads with fatigue).
2. All tested materials showed higher failure loads (>1750 N) than normal physiological occlusal loads in the posterior region (200–900N) and can be recommended for clinical use.
3. Irrespective of the material used in monolithic application, zirconia (Z-HT, Z-ST, Z-XT) and PICN restorations failed mostly from bulk fractures. When PICN was used as a table top, fracture was limited to PICN, whereas the 3Y-TZP framework remained intact.
4. PICN materials, especially in the proposed rapid-layer design, might be an interesting restorative treatment concept for zirconia implants due to their facilitated replaceability.

## Figures and Tables

**Table 1:** Restorative materials with group codes and investigated implant with characteristic flexural strength according to manufacturer.

Group (*n* = 24)	Crown/ Implant Design	Type	Name	Y_2_O_3_ (Weight%)	Flexural Strength (MPa)	Manufacturer
Z-HT	Monolithic Crown	3Y-TZP Zirconia	Vita YZ HT	4–6	1200	Vita Zahnfabrik, Bad Säckingen, Germany
Z-ST	Monolithic Crown	4Y-TZP Zirconia	Vita YZ ST	6–8	>850	Vita Zahnfabrik, Bad Säckingen, Germany
Z-XT	Monolithic Crown	5Y-TZP Zirconia	Vita YZ XT	8–10	>600	Vita Zahnfabrik, Bad Säckingen, Germany
E	Monolithic Crown	PICN	Vita Enamic	-	150–160	Vita Zahnfabrik, Bad Säckingen, Germany
RL	Rapid- Layer Crown	3Y-TZP Zirconia	Vita YZ HT	4–6	1200	Vita Zahnfabrik, Bad Säckingen, Germany
		PICN	Vita Enamic	-	150–160	
All groups	One-piece Implant	3Y-TZP Zirconia	ceramic. implant	5	1400	vitaclinical, Bad Säckingen, Germany

**Table 2:** Pretreatment and adhesive cementation of tested materials.

	Zirconia Crowns	Polymer-Infiltrated Ceramic Crown/Table Top	Zirconia Implant
Group	Z-HT, Z-ST, Z-XT, RL (framework)	E, RL	All
Surface Treatment	Air-particle abrasion with 50 μm Al_2_O_3_ at 2 bar, Ultrasonic cleaning with 70% ethanol for 3 min	Cleaning with 70% Ethanol, Etching with 5% hydrofluoric acid for 60 s (Vita Ceramics Etch, Vita Zahnfabrik), rinsed with air–water spray (30 s), ultrasonic cleaning with distilled water (5 min)	-
Primer	Clearfil Ceramic Primer Plus (Kuraray Noritake)		
Adhesive Cement	Panavia V5 opaque (Kuraray Noritake)		

**Table 3:** Descriptive statistics of failure load (in N). Statistically significant differences (*p* < 0.05) are indicated by different superscript letters within a column without (small letter) and with (capital letter) fatigue exposure. *t*- and *p*-values of t-tests for influence of fatigue by pairwise material comparison (within a row) (* indicates significance after Bonferroni correction: *p* < 0.01).

Group	Without Fatigue	With Fatigue	Influence of Fatigue	
	Mean ± SD	Mean ± SD	t-Value	*p*-Value
Z-HT	8450 ± 1443 ^a^	8145 ± 1426 ^A^	0.522	0.607
Z-ST	8161 ± 1024 ^a^	8557 ± 1288 ^A^	−0.833	0.414
Z-XT	6747 ± 795 ^b^	6110 ± 683 ^B^	2.103	0.047
E	1889 ± 278 ^c^	1769 ± 413 ^C^	0.834	0.413
RL	3938 ± 795 ^d^	5881 ± 862 ^B^	−5.736	<0.001 *

## Data Availability

The data presented in this study are available on request from the corresponding author.
